# Breaking stereotypes or making compromises? gender, income, and the unequal landscape of medical specialty choice

**DOI:** 10.1186/s12909-026-09797-1

**Published:** 2026-06-29

**Authors:** Ngoc Luong Khanh Nguyen, Hoang Viet Nguyen, Duong Thi Dang Ngo, Dung Boi Ly, Thuy Minh Ha, Quang Thanh Nguyen

**Affiliations:** 1 College of Health Sciences, VinUniversity, Hanoi Vietnam; 2 Department of Surgery, Vietnam National Children’s Hospital, Hanoi, Vietnam; 3 Innovations in Health Sciences, VinUniversity, Hanoi Vietnam; 4 Center for Medical and Health Sciences Education, University of Auckland, Auckland, New Zealand; 5 Royal College of Surgeons in Ireland and University College Dublin Malaysia Campus, George Town, Malaysia

**Keywords:** Gender, Income, Medical students, Medical education, Vietnam, Mixed method study, Specialty selection

## Abstract

**Background:**

Medical specialty choice shapes both physician identity and healthcare systems. Specialty choices are influenced not only by academic interest and aptitude but also by gender norms and socioeconomic pressures. While these dynamics are well documented in high income settings, fewer studies examine how perceived gender roles and income expectations jointly influence specialty preferences in LMICs contexts.

**Methods:**

This sequential mixed methods study combined a cross-sectional online survey with semi structured interviews. The quantitative phase included 155 medical students (response rate 79.5%) and examined associations between demographic characteristics and specialty preferences. The qualitative phase comprised 34 interviews purposively sampled across all five cohorts. Interview data were analyzed thematically using Braun and Clarke’s six step framework, following an inductive and reflexive approach guided by Social Cognitive Career Theory to explore how students understood and negotiated gender norms, income potential, and workload intensity.

**Results:**

Quantitative analysis showed no statistically significant associations between income-based specialty preference and gender, GPA, family income, hometown, or high school background (all *p* > 0.05). High income specialties were selected by 60 students (39%), low-income specialties by 26 (17%), and other fields by 69 (44%), with similar gender distribution across these groups (*p* = 0.81). In contrast, qualitative analysis revealed pronounced gendered patterns. Many female students chose specialties they perceived as low intensity, often citing anticipated caregiving responsibilities and work life balance concerns as reasons for avoiding surgery, emergency medicine, and other high intensity fields. Male students frequently reported social and familial pressure to pursue prestigious or high-income specialties, prioritizing financial stability and provider roles even when these conflicted with personal interests. Across both genders, themes highlighted the centrality of perceived income, prestige, mentorship inequities, and cultural narratives of sacrifice in shaping career compromises.

**Conclusion:**

Specialty choice among medical students in LMICs reflects not only personal interest but also structural inequities linked to gender norms and income expectations. Although quantitative analysis of demographic factors did not independently predict specialty preferences, qualitative findings revealed powerful social pressures that shape career compromises. Targeted, gender sensitive career counselling, equitable mentorship, and financial support are needed to enable students to choose specialties that align with their values and competencies.

**Supplementary Information:**

The online version contains supplementary material available at https://doi.org/10.1186/s12909-026-09797-1.

## Background

Medical specialty choice is a pivotal decision that shapes a physician’s professional identity, clinical focus, and long-term career trajectory [1]. It also carries important implications for health system functioning, workforce planning, and equitable access to care [1–3]. While personal interest and academic aptitude remain central, there are other factors that influence specialty choice, such as exposure to specialties during medical clerkships, mentorships, and sponsorships [1]. In addition, a growing body of evidence highlights the influence of socio demographic factors, particularly gender norms and perceptions of income potential, on specialty decision making [4–6].

Specialty choice is embedded within a complex sociocultural and economic environment. Medical students frequently negotiate competing pressures, balancing individual aspirations with cultural expectations, family obligations, and financial constraints [7, 8]. Gender roles often channel students toward or away from specific disciplines: surgical fields are frequently masculinized, whereas primary care and pediatrics are more commonly associated with women [9–11]. Anticipated income also plays a decisive role, particularly for students from lower-income households, who may feel compelled to prioritize financial stability and social mobility over personal interest or perceived societal need [12, 13]. Similar patterns have been observed in high income countries, where female students often favor controllable lifestyle specialties such as pediatrics or general practice, male students are more likely to choose high prestige fields such as surgery and radiology, and income and lifestyle expectations strongly shape perceived options [14–17].

Although interest in these issues is increasing, much of the existing literature originates from high income settings and may not fully capture the layered constraints that shape specialty choice in LMICs contexts. Recent work in Vietnam has begun to address this gap, showing how gendered expectations and economic pressures influence students’ perceptions of “appropriate” specialties and contribute to gender-based segmentation across disciplines [18]. Other studies have demonstrated that students’ ideals of what constitutes a “good doctor” are informed by culturally embedded gender narratives [1], and that current career counselling initiatives may be insufficient to counteract structural and cultural forces that steer students’ choices [3]. However, there remains limited empirical examination of how perceptions of gender and income interact, rather than operate in isolation, in shaping medical students’ specialty preferences.

To provide a coherent explanatory lens for these intertwined influences, the present study is guided by Social Cognitive Career Theory (SCCT). SCCT conceptualizes career choice as emerging from the dynamic interaction of self-efficacy beliefs, outcome expectations, personal goals, and contextual supports and barriers [19]. In the context of medical specialty selection, self-efficacy relates to students’ confidence in their capacity to succeed in specific fields. Outcome expectations encompass anticipated income, lifestyle, prestige, and family approval, and contextual factors include gender norms, family expectations, institutional structures, and labor market conditions. This framework is particularly suited to an LMICs context, where structural constraints and cultural scripts can strongly shape, and sometimes limit, the range of specialties that students view as attainable or appropriate [1, 3, 18].

### Study objectives

This study aims to investigate how medical students perceive the roles of gender norms and income potential in shaping their specialty preferences. It specifically addresses two research questions:

1. How do gender norms and gender stereotypes influence medical students’ specialty preferences and perceptions of specialty suitability and workload intensity, and how do these influences differ between female and male students?
2. How does the perceived income potential of different specialties influence students’ career decisions?

By addressing these questions, the study seeks to generate a contextually grounded understanding of how social and economic forces interact with students’ beliefs and goals to shape specialty choice in a LMICs setting.

## Method

### Ethical approval and study design

The study was conducted at VinUniversity, Vietnam, in December 2024. Ethical approval for the broader multifactorial project on medical students’ specialty choice was obtained from the VinUniversity Ethics Committee (Approval No. 55/2024/QDVMEC, dated 21 June 2024). The present manuscript reports a focused component of this project examining the influence of gender and income on specialty choice. As this component was also undertaken as part of a Master’s programme in Psychology at the University of Portsmouth, the questionnaire and study procedures were additionally approved by the Undergraduate and Taught Postgraduate Psychology Department Research Ethics Committee, University of Portsmouth (Approval No. 2024053, dated 14 November 2024). The study was conducted in accordance with the Declaration of Helsinki, and written informed consent was obtained from all participants.

The study used a sequential mixed methods design in which an initial quantitative survey was followed by a qualitative interview phase. The overall study design and integration of the two phases were informed by Social Cognitive Career Theory (SCCT), which guided the alignment of survey content, interview questions, and analytic focus with the core constructs of self-efficacy, outcome expectations, personal goals, and contextual supports and barriers [19]. This design was consistent with the explanatory sequential approach described by Creswell and Plano Clark [20]. The reporting of the qualitative component was informed by the Consolidated Criteria for Reporting Qualitative Research (COREQ) [21].

### Study setting, population, and recruitment

For the quantitative phase, the survey link was distributed through institutional email accounts, the university internal communication system, and class representatives, who shared it with peers in each cohort to maximize reach. Invitations were sent at the start of the data collection period, and reminder messages were sent once weekly during the survey window. For the qualitative phase, purposive sampling was used to ensure diversity in gender, academic performance, and specialty interest, with participants recruited from across all MD cohorts so that all academic years were represented. Eligible participants for interview were identified from survey respondents who indicated willingness to be contacted and were approached by face-to-face invitation. A total of 40 students were invited to participate in interviews, of whom 34 agreed and completed interviews. The complete questionnaire and interview guide are provided in the Supplementary Materials. Both instruments were developed through an extensive review of the literature on specialty choice and from previous work in the same setting, using established and previously used question formats [1, 3, 18].

### Research team and reflexivity

The qualitative phase aimed to deepen and contextualize the survey findings by exploring how students understood and negotiated gender norms, income expectations, and workload intensity when choosing specialties. Semi structured interviews were informed by a focused review of the literature on medical students’ specialty choice, gender norms, and financial considerations. The interview guide covered five interrelated domains: perceived income differences between specialties; gendered perceptions of specialties and expectations about appropriate roles for women and men; prestige, lifestyle, perceived barriers and facilitators to choosing particular specialties; and the influence of personal and social identity factors such as family background, values, and role models. During interviews, students were invited to describe which specialties they personally viewed as high income or low income, and as high intensity or low intensity in terms of clinical workload, emotional demands, and working hours. The research team did not provide predefined lists or definitions for these categories. Instead, income based and intensity-based classifications were generated inductively from participant accounts and were treated as perception based rather than as objective rankings of salary or workload. Students could also indicate that a specialty was not relevant to income-based classification, intensity-based classification, or both. The interview guide was pilot tested with 5 students, not included in the final sample, after which minor revisions were made to improve question clarity and flow.

Interviews were conducted in English or Vietnamese according to participant preference, either in a private room on campus or via a secure institutional videoconferencing platform. The interview team consisted of three fifth year and one fourth year medical students (two male and two female), all of whom had previous formal training and experience in qualitative research. No prior personal relationship was established between the researchers and participants before study commencement. Two clinician educators (TH and QN), both medical doctors with formal qualifications in health professions education, were affiliated with the same institution and provided training, supervision, and ongoing guidance for the interview process but did not directly interview participants. None of the interviewers were responsible for the formal assessment of the participating students, which helped to minimize perceived power differences. All members of the research team were based at the same medical school and shared a strong interest in understanding how gender norms, income expectations, and workload intensity influence specialty choice; this positionality was discussed in reflexive team meetings throughout the study. Each interview lasted approximately 30 to 45 min, was audio recorded with written informed consent, and transcribed verbatim by trained research assistants. No one other than the participant and interviewer was present during the interviews, no repeat interviews were conducted, and no field notes were made during or after the interviews. Vietnamese interviews were transcribed in the original language and then translated into English, and bilingual members of the team reviewed the translations to ensure accuracy.

All transcripts were deidentified and assigned study codes before analysis. As a form of member checking, a subset of participants (eight students) was invited to review either their individual transcript or a brief summary of preliminary interpretations and to comment on whether these reflected their intended meanings. Feedback from these participants was used to correct any factual inaccuracies and to refine the wording of themes and illustrative quotations, thereby enhancing the credibility of the qualitative findings.

### Data analysis

Quantitative data were analyzed using R Statistical Software (version 4.4.0). Categorical variables were summarized as frequencies and percentages, and continuous variables as means and standard deviations. Group differences were examined using Wilcoxon rank sum tests for two group comparisons and Kruskal Wallis tests for comparisons involving more than two groups. A significant level of *p* < 0.05 was applied throughout.

Qualitative data were analyzed thematically using Braun and Clarke’s six step framework, following an inductive and reflexive approach [22]. After repeated readings of the transcripts to achieve familiarization, initial codes were generated line by line and iteratively refined into broader categories. Two analysts, Dr QN and NN (a year 4 medical student with a degree in psychology and prior formal training in qualitative research), coded all transcripts independently using a shared codebook that was developed from an initial subset of interviews. Qualitative data management and coding were conducted using manual coding in Word and Excel. Any discrepancies in coding were discussed in analytic meetings and, when agreement was not reached, resolved by a third analyst, Dr TH, who has formal training in health professions education. Related codes were then grouped into overarching themes and subthemes through team discussion. Throughout the analytic process, the team engaged in reflexive consideration of their roles as educators and researchers within the same institution and how this positionality might influence interpretation. Thematic saturation was monitored and considered reached when no new codes or themes appeared in later interviews and existing themes were well supported by multiple participants.

## Result

Of the 195 eligible MD students, 155 completed the survey (response rate 79.5%). The sample included 75 female students (48.4%) and 80 male students (51.6%), with most respondents from urban hometowns and having attended competitive regional, top ranked public, or private and international high schools. For the qualitative phase, 34 students (19 females and 15 males) participated in semistructured interviews.

### Income and intensity-based classification of specialties

Although the qualitative phase followed the survey, the income and intensity categories derived from interview data were used during analysis to classify survey reported specialty preferences into income based and intensity-based brackets for integrated interpretation across both phases. Specialty categorization in this study was informed by students’ own perceptions, as elicited during qualitative interviews. Rather than imposing predefined categories, we allowed participants to identify specialties they regarded as high or low in terms of income and intensity, allowing for a student-driven, contextually grounded categorization. Specialties not explicitly mentioned were grouped under the category “Others”. Based on students’ own classifications, specialties most frequently identified as high income included plastic surgery, dermatology, surgery, and cardiology, which were associated with higher earnings, opportunities for private practice, and quicker returns on educational investment.

In contrast, fields such as pediatrics and family medicine were often described as lower income and less financially attractive, despite their central role in patient care. For perceived intensity, surgery, emergency medicine, and orthopedics were consistently placed in the high intensity group, characterized by long working hours, technical demands, and high stress, whereas specialties such as dermatology, radiology, psychiatry, pathology, rehabilitation, and family medicine were generally viewed as lower intensity because of more predictable schedules and better work life balance. Some specialties occupied mixed positions across the two dimensions: for example, dermatology and plastic surgery were often seen as both high income and low intensity, while obstetrics and gynecology was commonly regarded as high income but also demanding in terms of intensity. These patterns indicate that students distinguished income and workload as separate attributes of specialties, even though individual classifications varied for a small number of borderline fields. number of borderline fields.

### Quantitative findings

Table 1 compares demographic characteristics across students preferring high-income, low-income, and other specialties, revealing no statistically significant differences in gender, hometown, high school type, GPA, or family income. Gender distribution was balanced (*p* = 0.81), most students were from urban areas, and high school background showed no clear pattern (*p* = 0.36). While a slightly higher proportion of students in the low-income group had top GPAs, this was not significant (*p* = 0.26), nor were income differences (> 0.9). Overall, demographic variables did not independently predict specialty income preference.

**Table 1:** Demographic Characteristics by Specialty Income Preference Among Medical Students

Factors	High Income Specialties, (*n* = 60)	Low Income Specialties, (*n* = 26)	Others, (*n* = 69)	*p*-value^1^
Gender				0.81
Female	29 (48%)	14 (54%)	32 (46%)	
Male	31 (52%)	12 (46%)	37 (54%)	
Hometown				0.61
Rural	9 (15%)	6 (23%)	14 (20%)	
Urban	51 (85%)	20 (77%)	55 (80%)	
Highschool attended				0.36
Competitive Regional High Schools	10 (17%)	6 (23%)	16 (23%)	
Private/International High Schools	17 (28%)	12 (46%)	19 (28%)	
Standard Public High Schools	9 (15%)	1 (3.8%)	6 (8.7%)	
Top-ranked Public High Schools	24 (40%)	7 (27%)	28 (41%)	
Cumulative GPA				0.29
<2.00	1 (1.9%)	0 (0%)	2 (3.1%)	
2.00 to 2.49	1 (1.9%)	2 (8.7%)	4 (6.3%)	
2.50 to 3.19	15 (28%)	4 (17%)	23 (36%)	
3.20 to 3.59	30 (56%)	11 (48%)	23 (36%)	
3.60 to 4.00	7 (13%)	6 (26%)	12 (19%)	
Undisclosed	6 (10%)	3 (12%)	5 (7.2%)	
Monthly Family Income in Vietnamese Dong (VND)				> 0.9
< 30 million VND	19 (36%)	7 (32%)	20 (34%)	
30–50 million VND	10 (19%)	6 (27%)	11 (19%)	
> 50 million VND	24 (45%)	9 (41%)	28 (47%)	
Undisclosed	7 (12%)	4 (15%)	10 (14%)	

^1^Pearson’s Chi-squared test; Fisher’s exact test

**Fig. 1:**
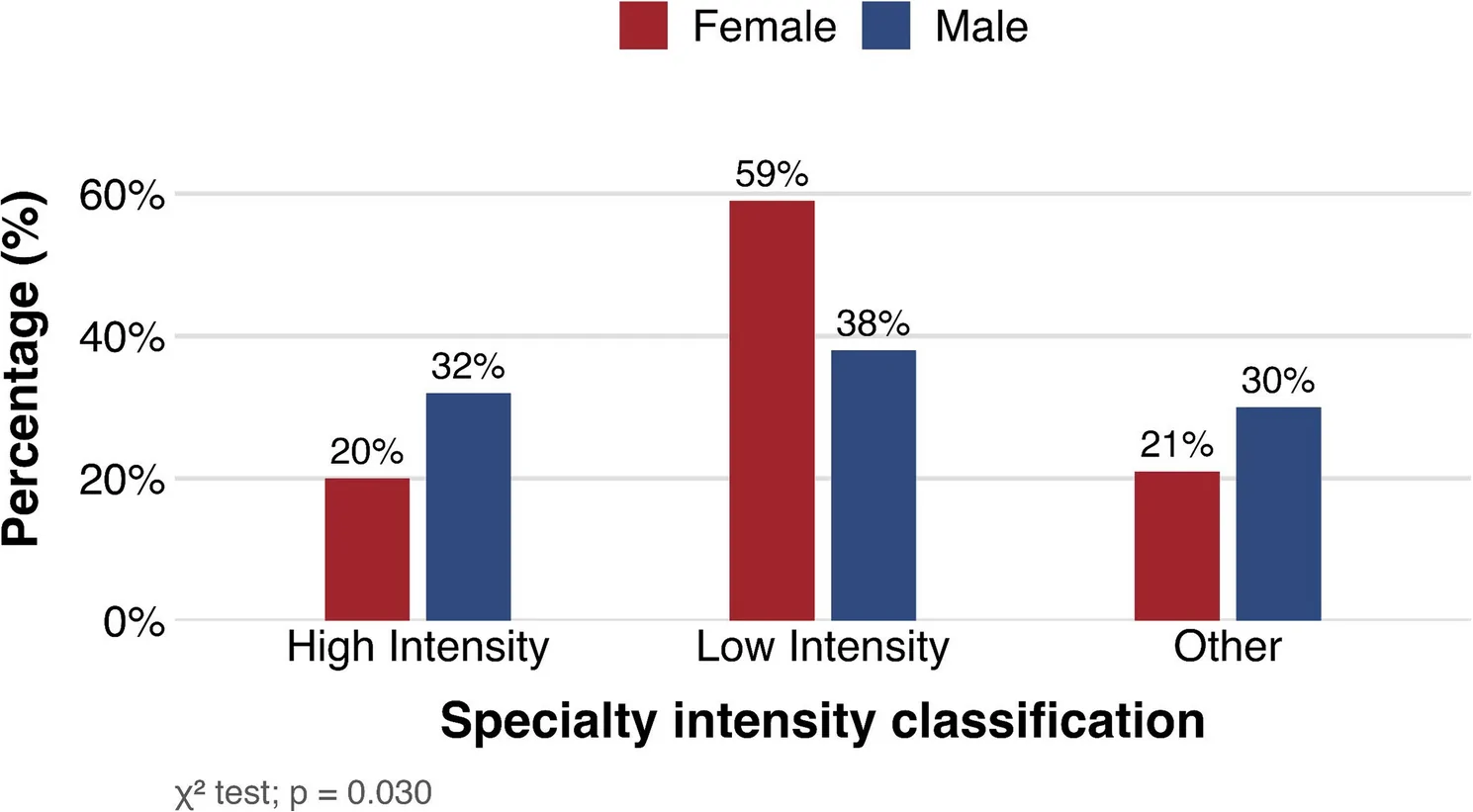
Distribution of selections of high intensity and low intensity medical specialties by gender

Figure 1 illustrates gender-based differences in preferred specialty intensity among medical students. A significantly higher proportion of female students (59%) opted for low-intensity specialties, compared to only 38% of male students. These findings suggest that perceived workload or lifestyle demands may differentially influence specialty preferences across genders, with female students tending to favor specialties perceived as less intensive.

### Qualitative findings

Thematic analysis generated four overarching themes with related subthemes: (1) Income-Driven Decision-Making and the Prestige Hierarchy; (2) Gender Norms and Perceived Specialty Suitability; (3) Work–Life Balance and Gendered Career Planning; and (4) Structural Inequities and Sociocultural Gatekeeping.

### Theme 1: income-driven decision-making and the prestige hierarchy

#### Subtheme 1.1: high-income specialties and pragmatic motivations

Students consistently identified high-income specialties, such as plastic surgery, dermatology, and cardiology, as highly desirable. These fields were associated not only with financial gain but also with autonomy, private practice opportunities, and quicker returns on educational investment.

“I think if I choose plastic surgery or dermatology, I’ll be able to work independently and earn well. Especially in Vietnam, people pay for beauty services without insurance.” (Female, Year 2)

This quote reflects students’ awareness of Vietnam’s private healthcare economy, where income is often linked to market-driven services rather than solely to clinical workload. Among students from lower socioeconomic backgrounds, the motivation to relieve financial pressures on their families emerged repeatedly, making income a dominant factor in their decision-making:

“My family has supported me a lot. I want to pay them back soon. That’s why I cannot go for something too long or low-paid.” (Male, Year 4)

In such cases, specialty selection transcended personal interest, taking on the form of a moral obligation shaped by familial duty, a finding particularly relevant in collectivist cultures like Vietnam.

#### Subtheme 1.2: prestige as social capital

Prestige, though distinct from income, was often discussed in tandem. Specialties such as surgery and cardiology were highly regarded as prestigious due to their complexity and direct involvement in life-saving interventions. Students described these specialties as “elite,” conferring symbolic capital that could enhance social standing.

“Even if dermatology pays more, it doesn’t feel as prestigious as being a surgeon or a cardiologist. Society respects those who deal with life and death.” (Male, Year 3)

This reflects an underlying symbolic hierarchy: prestige is awarded based on perceived difficulty, intensity, and societal impact. Notably, some students expressed conflict between the desire for prestige and practical constraints such as training duration and resource availability.

“I want to become a neurologist, but it’s competitive and takes many years. Maybe I should pick something simpler even if I don’t admire it as much.” (Female, Year 4)

Such tensions were particularly pronounced among students lacking financial support or access to mentorship, revealing that prestige can become an inaccessible ideal rather than a realistic goal.

### Theme 2: gender norms and perceived specialty suitability

#### Subtheme 2.1: gendered expectations and specialty pathways

Many participants reported that gender stereotypes shaped perceptions of which specialties were suitable for men and women. Surgery, emergency medicine, and orthopedics were described as masculine domains that were seen to require physical stamina, decisiveness, and leadership, qualities that participants felt were commonly attributed to men in Vietnamese society.

“People assume men are tougher, so they belong in surgery. When a girl says she wants to be a surgeon, people look surprised.” (Female, Year 1)

Female students frequently mentioned being steered, sometimes subtly and sometimes overtly, toward “softer” fields such as pediatrics, dermatology, and obstetrics, particularly by relatives and senior doctors.

“My aunt is a doctor and told me surgery is not for women. Too demanding, too tiring, and you will not have time for a family.” (Female, Year 2)

Similar comments were reported across multiple interviews, indicating that advice about specialty choice was often communicated in gendered terms.

#### Subtheme 2.2: patient preferences and gender advantage

While gender bias often posed barriers for women in high-intensity fields, it offered certain advantages in female-focused care. Many female students described being encouraged to enter obstetrics and gynecology because of patient preferences.

“Patients feel more comfortable with female doctors, especially in OB-GYN. So it’s seen as a good choice for us.” (Female, Year 5)

However, this perceived advantage is double-edged. Male students expressed hesitation about entering such specialties, fearing stigma or reduced opportunities.

“I like OB-GYN, but some people say it’s strange for a man to choose that. It feels like I won’t be welcomed.” (Male, Year 1)

These dynamics reinforce occupational segregation along gender lines, driven not only by societal norms but also by assumptions within clinical settings and patient interactions.

### Theme 3: work-life balance and gendered career planning

#### Subtheme 3.1: familial roles shaping female decision-making

A recurring theme among female students was the need to anticipate future family responsibilities when selecting a specialty. These concerns were not hypothetical, but shaped by familial advice, observations of female doctors’ experiences, and societal discourse around ideal womanhood.

“I always hear that being a good mother means being present. If I become a surgeon, I won’t be home. People will judge me.” (Female, Year 4)

This internalization of traditional gender roles often led female students to prioritize specialties with predictable schedules—even at the cost of lower income or professional recognition.

“Even though I’m passionate about emergency medicine, I know I won’t have time for my kids. So I’m thinking about dermatology.” (Female, Year 2)

#### Subtheme 3.2: male pressure to prioritize income over interest

In contrast, male students described external pressure to pursue high-income or prestigious specialties, regardless of personal interest. For them, deviation from this path was perceived as a failure to fulfill expected masculine roles tied to ambition, responsibility, and financial success.

“Sometimes I wonder if I’d be happier in a calmer field, but then I feel guilty. I’m supposed to aim high and earn well.” (Male, Year 3)

For male students, work-life balance was less about caregiving and more about long-term financial planning and social standing. The role of provider was strongly internalized, reinforcing the economic rationale behind their specialty choices.

### Theme 4: structural inequities and sociocultural gatekeeping

#### Subtheme 4.1: uneven mentorship and gender bias in clinical exposure

Mentorship was reported as inconsistent and, at times, biased. Several female students noted that male peers were more frequently invited to assist in clinical procedures, particularly in surgical rotations, reinforcing gender disparities in skill development and confidence.

“In surgery, teachers call male students to help. We’re told to observe or stay outside. How can we learn that way?” (Female, Year 2)

The effect of such inequity is cumulative. Fewer procedural experiences early in training lead to reduced confidence and fewer residency applications in surgical fields by female students.

#### Subtheme 4.2: cultural narratives of sacrifice and success

Students of all genders described narratives of sacrifice and duty embedded in the medical profession, but the cultural interpretation of such sacrifice varied by gender. A man who sacrifices time and health for his career is admired; a woman who does the same is often questioned.

“When a man stays late, people say he’s dedicated. When a woman does it, they ask about her children.” (Female, Year 4)

These cultural scripts discourage women from aspiring to “heroic” high-intensity professional identities and penalize them socially for ambition. For male students from low-income families, however, these narratives also come with pressure to succeed at all costs, often without adequate institutional support.

“I want to become a neurosurgeon, but the training is long, and I can’t afford the exam fees. It feels like only rich students can make it.” (Male, Year 3)

Thus, income and gender intersect with class and cultural expectations to create layered inequities in access to high-prestige, high-income specialties.

Taken together, these findings reveal a complex landscape in which gendered expectations, perceived income hierarchies, and socioeconomic pressures intersect with cultural and institutional structures to shape students’ specialty decision-making. The themes highlight that specialty choice emerges not as a purely rational process, but as one embedded in identity work, structural inequality, and emotional negotiation.

## Discussion

Drawing on Social Cognitive Career Theory (SCCT), our findings show how gender norms, income expectations, and structural influences interact to shape medical students’ specialty preferences. The results illustrate that career choices arise from an interplay of beliefs about one’s capability, anticipated income and lifestyle, evolving career goals, and perceived supports and barriers [19]. In this setting, perceived gender roles, socioeconomic position, and institutional structures jointly shaped students’ aspirations in ways that were often complex and at times internally contradictory.

Self-efficacy, understood as students’ confidence in their ability to succeed in a given specialty, appeared strongly gendered in both expression and development. Female students were less likely to view themselves as suitable for high intensity specialties such as surgery or emergency medicine, even when they demonstrated academic and clinical competence. They frequently attributed this hesitation to internalised stereotypes and the lack of visible female role models in male dominated fields, echoing broader evidence on gendered expectations in medicine [23]. Male students, by contrast, more often expressed confidence in pursuing high status specialties irrespective of workload, with this confidence reinforced by societal narratives that celebrate and reinforce men’s authority and technical capability [24]. These patterns illustrate how SCCT constructs of self-efficacy are embedded within gendered social contexts rather than being purely individual attributes.

Outcome expectations, particularly regarding income and lifestyle compatibility, also played a central role. High income specialties were widely associated with financial security, professional prestige, and family approval, especially among students from lower income households. At the same time, many female students emphasised anticipated work life balance and future caregiving responsibilities, which led them to view some high intensity, high income specialties as misaligned with their long-term goals [25]. Importantly, outcome expectations were not static. Students described how, over time, their priorities shifted from extrinsic outcomes such as salary and prestige toward intrinsic goals that emphasised fulfilment, patient relationships, and meaningful work. This evolution mirrors longitudinal evidence that medical students often move from extrinsic to intrinsic motivations as training progresses [26, 27].

Personal goals and emerging professional identities were frequently reshaped through clinical exposure and mentorship. Several students who initially intended to pursue high income specialties described changing course after positive experiences in fields such as paediatrics or family medicine. These shifts underscore the importance of authentic clinical experiences and reflective learning in shaping specialty preferences. They also align with the concept of person environment fit, which posits that students seek careers that match their values, interests, and personal characteristics [28]. Students who strongly valued empathy, communication, and relational work tended to favour primary care or paediatrics, whereas those who perceived themselves as more tolerant of stress or attracted to high acuity decision making tended to prefer surgical or other high intensity disciplines.

Contextual and structural factors further mediated these individual level processes. Students from rural areas or lower socioeconomic backgrounds often foregrounded the cost of postgraduate training, duration of specialisation, and uncertainty in the job market. For these students, specialty choice was commonly framed in terms of financial responsibility to their families. Some male students described a perceived obligation to become the primary income earner, which drew them toward specialties considered financially rewarding. In contrast, female students frequently encountered subtle discouragement from pursuing high stakes or time intensive careers due to anticipated conflicts with future family responsibilities. These findings echo literature on gender role conflict, emotional labour, and the disproportionate burden of caregiving expectations placed on women in medicine [9, 29].

Cultural expectations amplified these dynamics. In Vietnam, traditional values informed by Confucian ideals continue to influence what is considered appropriate work for men and women [30]. Female students reported being steered toward specialties associated with caregiving and emotional labour, while male students were encouraged to pursue prestigious and financially secure careers. Some female students expressed clear interest in surgery or other male dominated fields but described these paths as incompatible with femininity, motherhood, or broader social acceptance. Financial background further shaped how students navigated trade-offs. Students from more economically secure families reported greater freedom to choose specialties based on interest and values, even when these choices were not financially optimal, whereas those under financial strain were more likely to prioritise economic stability. Yet, consistent with Philip et al. [31], the relationship between income and specialty choice in this study was nuanced and context dependent rather than strictly deterministic, with many students actively weighing long term quality of life and emotional wellbeing against immediate financial gain.

Institutional hierarchies and patterns of exposure also influenced what students perceived as realistic or aspirational. Limited contact with diverse role models, particularly female leaders in high intensity specialties or male physicians working in primary care, narrowed the perceived scope of possible careers [32]. Curricular emphasis on certain disciplines, along with the relative visibility of specific departments, reinforced prestige-based hierarchies that positioned some specialties as default aspirations and others as secondary options. Within the SCCT framework, these institutional features can be understood as contextual affordances and barriers that shape both self-efficacy and outcome expectations.

The patterns of findings from LMICs discussed above resonate with and extend those reported in high income settings. Studies from Sweden, the Netherlands, and France show that female students frequently prioritise work life balance and controllable lifestyle specialties, such as paediatrics or general practice, and may see themselves as less suited to high intensity fields, in part due to internalised stereotypes and limited female role models [14, 33, 34]. Male students in these contexts are often drawn to high income, high prestige specialties such as surgery or radiology, consistent with observations that male confidence and aspirations are reinforced by narratives of authority and technical skill [15, 35]. Outcome expectations related to income and lifestyle similarly shape choices in high income countries, where high earning specialties are commonly linked with financial security and status [16], while women’s concerns about lifestyle and caregiving responsibilities influence both entry into and persistence within demanding fields such as neurosurgery [36–38]. Evidence from Yang et al. [27] further suggests that academic interests and intrinsic motivations may play an increasingly prominent role in specialty decisions in high income settings, complementing the patterns observed here.

Socioeconomic context and institutional structures also exert measurable influence across settings. In the United Kingdom, students from less advantaged backgrounds appear more inclined toward community-oriented careers and less competitive specialties, suggesting that financial background shapes perceived options and acceptable trade-offs in ways that parallel the present findings [12, 17]. Persistent gender discrimination and subtle bias, as documented among female medical students in Germany, contribute to a hidden curriculum that discourages women from particular specialties and reinforces structural inequities [39]. Taken together, these international comparisons suggest that while cultural and system level nuances differ, the core interplay of gender norms, financial considerations, and institutional factors is a consistent feature of medical career decision making across both LMICs and high-income contexts.

### Recommendations arising from study findings

1. Address gender stereotypes in specialty selection

This study highlights the persistent influence of gender norms on specialty choices, with female students often guided toward caregiving, lower intensity fields and discouraged from entering surgery or other high intensity specialties. To counteract these patterns, medical schools should incorporate training on unconscious bias and gender equity into both student curricula and faculty development [40]. Expanding access to diverse mentors, particularly women in leadership roles within traditionally male dominated specialties, can broaden students perceived possibilities and challenge internalized assumptions [40].

2. Promote strategic financial support for undervalued specialties

Financial concerns, especially among students from lower income families, directed some participants toward higher income specialties. Institutions should develop targeted financial support for students pursuing fields such as family medicine, geriatrics, and pediatrics. Providing full tuition and fee scholarships can allow students to choose a specialty with less pressure from educational debt [41]. For example, the Abigail Geisinger Scholars Program offers full tuition, fees, and a living stipend to students who commit to working in primary care or psychiatry following residency, directly addressing critical workforce needs [42]. Financial stipends can also support participation in specialized summer immersion experiences designed to increase interest in fields such as child and adolescent psychiatry [43].

3. Strengthen and personalize career guidance

Although students recognized the value of career counselling, many felt that its influence on their decisions was limited. Medical schools should expand these services to provide early, longitudinal, and personalized guidance. Structured exposure to a wide range of specialties through shadowing, career seminars, mentorship, and reflective workshops can help students move beyond stereotyped images of specialties [1, 3, 18].

Tools informed by frameworks such as Person Environment Fit can help students align career paths with their personal values, working styles, and long-term goals. The Person Environment Fit framework suggests that positive outcomes, including satisfaction and wellbeing, are more likely when individuals work in environments that match their characteristics [44]. Integrating such tools into career counselling may support more reflective and sustainable specialty decisions.

4. Improve accessibility of high intensity specialties for all students

Concerns about the compatibility of high intensity specialties with family responsibilities, particularly among female students, frequently discouraged entry into these fields. A supportive work environment, characterized by understanding supervisors and flexible organizational structures such as adaptable work schedules, can mitigate work home tensions [45]. The implementation of implicit bias training increased institutional support, formal mentorship initiatives, early exposure programs, transparent promotion policies, childcare support, and accommodation of maternity leave are necessary to retain women in surgery [46]. Although surgical training programs are approaching gender parity, pregnancy and parenthood remain challenging due to inconsistent and brief parental leaves and limited postpartum support [46]. Medical students consider an institution’s parental leave policy an important factor when ranking residency programs [47]. Time constraints and career goals disproportionately affect female surgeons considering childbearing, underscoring the need for gender specific institutional resources and for addressing barriers to family planning [48].

Taken together, these recommendations aim to reduce structural and cultural barriers so that students can pursue specialties according to interest, aptitude, and values, rather than being constrained primarily by societal norms or financial pressures.

### Limitations of the study & future research

This study has several limitations that should be considered when interpreting the findings. First, the research was conducted at a single, newly established private medical school, which may limit the generalisability of the results. The cross-sectional design captures students’ views at one point in time and does not allow examination of how individual specialty preferences and perceptions change as students progress through training. Because specialty choice often evolves and is frequently finalized in more senior years after greater clinical exposure, including students in earlier years may have influenced the distribution of stated preferences and the salience of particular factors, and therefore the findings should be interpreted as reflecting specialty intentions rather than definitive career decisions. The study also relied on self-reported data, which may be affected by social desirability bias, especially for sensitive topics such as gender norms and income considerations.

A further limitation concerns the way specialties were classified as high income or low income and as high intensity or low intensity. These classifications were intentionally based on student perceptions rather than on objective salary or workload data, and national level data on specialty specific income and working conditions in Vietnam remain limited. As a result, the perceived categories used in this study may not fully align with actual income distributions or workload patterns across specialties. In addition, the sample included a high proportion of students from urban backgrounds and competitive, top ranked public, or private and international high schools, which may reflect a more socioeconomically advantaged student population and could be associated with higher family incomes; this may limit transferability to students in more rural settings.

Future research should therefore extend this work in several directions. Multicentre studies that include public and private institutions across different regions would help clarify how local sociocultural, economic, and institutional contexts shape both perceptions and choices. Longitudinal designs are needed to follow students over time and examine how gender norms, financial considerations, family expectations, and evolving personal values interact to influence specialty decisions at key transition points, such as entering clinical clerkships or applying for residency. Studies that integrate objective indicators, such as available income and workload data, with measures of student perceptions would also allow comparison between perceived and actual specialty characteristics.

## Conclusion

This study highlights the complex interplay between gender norms, income expectations, and structural influences in shaping medical students’ specialty preferences within a resource-constrained, culturally layered setting. While individual aspirations and personal interests remain important, students’ decisions are deeply informed by gendered expectations, perceived financial trajectories, and sociocultural obligations. Female students often face implicit discouragement from pursuing high-intensity or traditionally male-dominated specialties, whereas male students experience pressure to prioritize income and prestige over interest or well-being. Financial background further amplifies these dynamics, affecting perceived accessibility of certain specialties. By centering students’ voices and perceptions, this study contributes to a more nuanced understanding of career decision-making in medical education and underscores the need for institutional, cultural, and policy-level interventions to ensure equitable career development for future physicians.

## Supplementary Information

Supplementary Material 1.

## Data Availability

The datasets generated and/or analyzed during the current study are not publicly available due to privacy restrictions but are available from the corresponding author on reasonable request.
