# Supplementary material for: Breaking stereotypes or making compromises? gender, income, and the unequal landscape of medical specialty choice
Source: BMC Med Educ. 2026 Jun 29;26:1476. doi: 10.1186/s12909-026-09797-1 (PMC13584382; doi:10.1186/s12909-026-09797-1)
Supplement: Supplementary file 1 [file 12909_2026_9797_MOESM1_ESM.docx]

**SURVEY QUESTIONS AND INTERVIEW GUIDE**

**Exploring Perceptions of Income, Intensity, Gender, and Career Preferences Among Medical Students**

Dear Participant,

You are invited to participate in a research study exploring how medical students perceive income differences, workload intensity, gender roles, and specialty preferences within the medical profession. This study is conducted as part of a research project at VinUniversity.

**Purpose of the Study:** This study aims to better understand how medical students at different stages of their training perceive and make decisions about medical specialties, particularly in relation to income, work-life balance, gender, and prestige.

All responses will be kept strictly confidential. Data will be anonymized before analysis. No personally identifiable information will be disclosed or published.

**I. QUANTITATIVE**

1. **Cohort (Year in Medical School):**
    ☐ Year 1 ☐ Year 2 ☐ Year 3 ☐ Year 4 ☐ Year 5
2. **Age:**
    ____ years
3. **Gender:**
    ☐ Female ☐ Male ☐ Prefer not to say
4. **Place of Permanent Residence:**
    ☐ Urban area (e.g., Hanoi, Ho Chi Minh City)
    ☐ Rural or small towns
5. **High School Attended:**
    ☐ Competitive Regional High School
    ☐ Private or International High School
    ☐ Standard Public High School
    ☐ Top-ranked Public High School
6. **Cumulative GPA (as of most recent academic year):**
    ☐ <2.00
    ☐ 2.00 – 2.49
    ☐ 2.50 – 3.19
    ☐ 3.20 – 3.59
    ☐ 3.60 – 4.00
    ☐ Prefer not to say
7. **Average Monthly Family Income (in Vietnamese Dong):**
    ☐ < 30 million VND
    ☐ 30 – 50 million VND
    ☐ > 50 million VND
    ☐ Prefer not to say
8. **What is your current preferred specialty?**
    ☐ Surgery
    ☐ Dermatology
    ☐ Cardiology
    ☐ Emergency Medicine
    ☐ Pediatrics
    ☐ Family Medicine
    ☐ Internal Medicine
    ☐ Obstetrics and Gynecology
    ☐ Psychiatry
    ☐ Radiology
    ☐ Pathology
    ☐ Rehabilitation Medicine
    ☐ Public Health
    ☐ Other: ___________
    ☐ Not sure

**II. QUALITATIVE**

**Section A: Perceptions of Income Differences**

1. *In your opinion, which medical specialties offer the highest income?*
   → Follow-up: Why do you think those specialties are higher earning?
2. *Which specialties do you think offer the lowest income?*
   → Follow-up: What makes you think they are less financially rewarding?
3. *Do you think doctors in Vietnam are generally paid fairly for their work?*
   → Follow-up: Do you feel any specialty is particularly underpaid or overpaid?
   → Prompt if needed: What do you think about pay in family medicine or internal medicine?

**Section B: Intensity, Lifestyle, and Prestige**

4. *Which specialties do you consider to be the most intense or stressful?*
   → Follow-up: What factors (e.g., hours, workload, procedures) influence your judgment?
5. *Which specialties seem to offer better work-life balance or predictability?*
   → Follow-up: Are there specialties you would avoid because of intensity?
6. *Do you feel that some specialties are seen as more prestigious than others?*
   → Follow-up: Why do you think that is?
   → Optional probe: How does prestige influence your own career thinking?
7. Can you describe any obstacles that you or your family have faced that may influence your choice of specialty? → Prompt: Financial hardship, geographic location, lack of connections, or other difficulties?

**Section C: Gender Norms and Career Pathways**

7. *Do you think some specialties are more suited to men or to women?*
   → Follow-up: Can you give examples? What shapes these ideas?
8. *Do you believe male and female doctors are treated equally in all specialties?*
   → Follow-up: Have you witnessed or heard examples of inequality?
9. *Do you think gender may affect job promotion or leadership opportunities in medicine?*
   → Follow-up: Would this influence your choice of specialty?
10. Is there anything else you would like to share about your career aspirations, experiences in medical school, or the factors influencing your specialty choice? Do you have any further thoughts on how gender and income may shape your decision?

Thank you very much for your participation.

Your input is valuable and will help inform improvements in medical education policy and support for future students.
